# The Role of Exosomes and Exosomal Noncoding RNAs From Different Cell Sources in Spinal Cord Injury

**DOI:** 10.3389/fncel.2022.882306

**Published:** 2022-04-18

**Authors:** Zhe-Lun Yang, Jian Rao, Fa-Bin Lin, Ze-Yan Liang, Xiong-Jie Xu, Yi-Ke Lin, Xin-Yao Chen, Chun-Hua Wang, Chun-Mei Chen

**Affiliations:** Department of Neurosurgery, Fujian Medical University Union Hospital, Fuzhou, China

**Keywords:** spinal cord injury, exosome, mesenchymal stem cell, neural stem cell, noncoding RNAs

## Abstract

Spinal cord injury (SCI) not only affects the quality of life of patients but also poses a heavy burden on their families. Therefore, it is essential to prevent the occurrence of SCI; for unpreventable SCI, it is critical to develop effective treatments. In recent years, various major breakthroughs have been made in cell therapy to protect and regenerate the damaged spinal cord *via* various mechanisms such as immune regulation, paracrine signaling, extracellular matrix (ECM) modification, and lost cell replacement. Nevertheless, many recent studies have shown that the cell therapy has many disadvantages, such as tumorigenicity, low survival rate, and immune rejection. Because of these disadvantages, the clinical application of cell therapy is limited. In recent years, the role of exosomes in various diseases and their therapeutic potential have attracted much attention. The same is true for exosomal noncoding RNAs (ncRNAs), which do not encode proteins but affect transcriptional and translational processes by targeting specific mRNAs. This review focuses on the mechanism of action of exosomes obtained from different cell sources in the treatment of SCI and the regulatory role and therapeutic potential of exosomal ncRNAs. This review also discusses the future opportunities and challenges, proposing that exosomes and exosomal ncRNAs might be promising tools for the treatment of SCI.

## Introduction

According to the etiology, spinal cord injury (SCI) can be divided into traumatic and nontraumatic. Traumatic SCI is often caused by severe damage to the spinal cord due to external physical impacts (for example, car accidents, falls, sports-related injuries, or violence). Nontraumatic SCI often occurs during acute or chronic diseases (for example, tumors, infections, disc herniation, or vertebral fracture-dislocations), causing spinal cord compression to produce primary and secondary injuries. SCI not only affects the quality of life of patients but also poses a heavy burden on their families. Therefore, it is essential to prevent the occurrence of SCI; for unpreventable SCI, it is critical to develop effective treatments (Ahuja et al., 2017).

A central concept in managing any SCI patient has been “time is the spine” (Ahuja et al., 2017). SCI is characterized by the progressive loss of neurologic function within a few hours. Therefore, it is essential to rapidly diagnose patients and provide neuroprotective interventions in the acute injury stage. Because of these treatments including hemodynamics (Ryken et al., 2013), hormonal therapy (Hurlbert et al., 2015), and surgical decompression (Ramakonar and Fehlings, 2021), long-term functional recovery is improved in patients. Management of patients with SCI is complex and involves multiple stages of care, often lasting several years after the initial injury. Thus, later rehabilitation is also an integral part of the treatment process (Gómara-Toldrà et al., 2014; van der Scheer et al., 2021).

However, these treatments are not adequate for long-term functional recovery in SCI patients. In recent years, various major breakthroughs have been made in cell therapy to protect and regenerate the damaged spinal cord *via* various mechanisms such as immune regulation, paracrine signaling, extracellular matrix (ECM) modification, and lost cell replacement. Among them, the most commonly studied and promising cell types include induced pluripotent stem cells (iPSC), mesenchymal stem cells (MSCs), neural stem cells (NSCs), oligodendrocyte progenitor cells (OPCs), Schwann cells (SCs), and olfactory ensheathing cells (OECs; Harrop et al., 2012; Shao et al., 2019; Ahuja et al., 2020). Nevertheless, many recent studies have shown that the cell therapy has many disadvantages, such as tumorigenicity, low survival rate, and immune rejection. Because of these disadvantages, the clinical application of cell therapy is limited (Feng et al., 2021; Liu J. et al., 2021).

The remarkable effect of cell therapy can be attributed to its dominant paracrine effect. Exosomes, an intercellular communication tool, affect normal and pathological conditions. In recent years, the role of exosomes in various diseases and their therapeutic potential have attracted much attention. The same is true for exosomal noncoding RNAs (ncRNAs), which do not encode proteins but affect transcriptional and translational processes by targeting specific mRNAs. Their diverse functions have attracted much interest (Colombo et al., 2014; Quinzaños-Fresnedo and Sahagún-Olmos, 2015; Shi et al., 2018; Dutta et al., 2021). The types of source cells can influence the heterogeneity of exosomes, which have different contents and specific markers from varied cell sources. For example, ERBB2 is specifically expressed in breast cancer cell-derived exosomes, and TSPAN8 is a specific marker of epithelial cell-derived exosomes. The inherent biology and the microenvironment of the cells can also give exosomes distinct functions such as uptake by specific cells and tropism to certain organs (Kalluri and LeBleu, 2020). Especially, exosomal miRNAs have attracted substantial attention because of their various functions in the context of SCI treatment. They vary widely with different cell sources (Cho et al., 2019). For example, neuron-derived exosomes were enriched for miRNA-383, whereas glial cells-derived exosomes were not (Pomper et al., 2020). It is noteworthy that a study directly compared the efficacy of human pluripotent stem cells (hPSCs)-derived and MSCs-derived exosomes in an animal model of ischemic stroke. Results showed that hPSCs-derived exosomes were more effective than MSCs-derived (Webb et al., 2018).

MSCs-derived exosomes are the most widely studied to date in the treatment of SCI. However, no studies have directly compared the efficacy of exosomes from different cell sources in the treatment of SCI (Dutta et al., 2021). Therefore, it is essential to directly compare the therapeutic potential of exosomes from varied cell sources in the treatment of SCI. This review focuses on the mechanism of action and therapeutic potential of exosomes and exosomal ncRNAs from different cell sources in the treatment of SCI. This review also discusses the future opportunities and challenges, proposing that exosomes and exosomal ncRNAs from different cell sources might be promising tools for the treatment of SCI.

## Pathophysiological Process of SCI

According to its pathological process, traumatic SCI has a complex pathophysiological process, and it can be broadly classified into primary and secondary injuries. The primary damage can immediately cause mechanical destruction and dislocation of the spine, causing spinal cord compression or transection. Subsequently, the primary injury leads to a continuous cascade of secondary injuries, causing further damage to the spinal cord and neurological dysfunction. Then, this injury causes damage to myelin, axons, and neurons. Moreover, it also disrupts the blood-spinal cord barrier. The primary damage is often irreversible, while the secondary injury usually causes more severe damage than the primary injury (Tator, 1995; McDonald and Sadowsky, 2002; Ahuja et al., 2017).

SCI is divided into five stages depending on the pathophysiological process and the time of injury: hyperacute phase (0–2 h), acute phase (2–48 h), subacute phase (2–14 days), intermediate phase (2 weeks to 6 months), and chronic phase (>6 months; Rowland et al., 2008). In the hyperacute phase (0–2 h), it is characterized by traumatic axons, hemorrhagic necrosis of gray matter, and microglial activation releasing proinflammatory cytokines. The proinflammatory cytokines TNFα and IL-1β can be immediately released by the microglial after injury (Donnelly and Popovich, 2008). In the acute phase (2–48 h), it is characterized by vasogenic and cytotoxic edema, persistent hemorrhage and necrosis of glutamate-mediated excitotoxicity, disruption of blood-spinal cord barrier (BSCB) permeability, early demyelination, axonal swelling, and neuronal death. The BSCB can remain disrupted even at 28 days after SCI and spread along the entire length of the cord (Whetstone et al., 2003). The complex network of tight junction (TJ) proteins, which are the major protein component of the BSCB, can be modulated by inflammatory cytokines (Lee et al., 2012; Kumar et al., 2017). In the subacute phase (2–14 days), it is characterized by persistent edema, thrombosis, and vasospasm aggravate ischemia. A continual inflammatory cell infiltration causes further cell death, forming cavities. In addition, reactive astrocytes act as a barrier to prevent damage from aggravating but secrete some inhibitory ECM molecules around the lesion. Chondroitin sulfate proteoglycans (CSPGs), which are a key component of the ECM, can inhibit axon regeneration through binding to their major cognate receptor, Protein Tyrosine Phosphatase Sigma (PTPσ; Sakamoto et al., 2019). Therefore, blocking the combination of them can effectively promote axonal regeneration. In the intermediate phase (2 weeks to 6 months), the axons continue to degenerate, and reactive astrocyte scars mature, becoming an effective regenerative inhibitor, and the cysts merge to limit axonal regeneration and cell migration. The scars have two distinct components after SCI: the lesion core, which primarily includes macrophages and fibroblasts, is generally considered as the fibrotic scar, and the lesion border, which is predominantly composed of microglia, reactive astrocytes, and NG2+ oligodendrocyte progenitor cells, is commonly regarded as a glial scar (Bradbury and Burnside, 2019; Tran et al., 2021). In the traditional concept, the glial scar is considered to exert a detrimental function for neurological recovery, which not only secretes some inhibitory extracellular matrix molecules, cytokines, and oxidative stress products but also inhibits axonal regeneration as a chemical barrier (Silver and Miller, 2004). However, many studies have confirmed that glial scar plays a vital role in neuroprotection. It can limit the spread of inflammation at the injury site to the surrounding injury as a barrier. And it can also secrete neurotrophic factors (nerve growth factor and fibroblast growth factor) and extracellular matrix proteins (laminin and fibronectin; Lukovic et al., 2015). Therefore, an increasing number of studies are focusing on the beneficial function of the glial scar for neurological recovery. In the chronic phase (>6 months), the Wallerian degeneration process of severed axons continues, and the severed axons and their cell bodies may take years to be completely removed (Ehlers, 2004). Unfortunately, neurological dysfunction and neuropathic pain will be caused by the formation of syringomyelia (Todor et al., 2000). Therefore, therapeutic strategies aim to improve axonal degeneration and demyelination by drug or cell transplantation.

## The Role of Exosomes and Exosomal ncRNAs from Different Cell Sources in The Treatment of SCI

**Figure 1:**
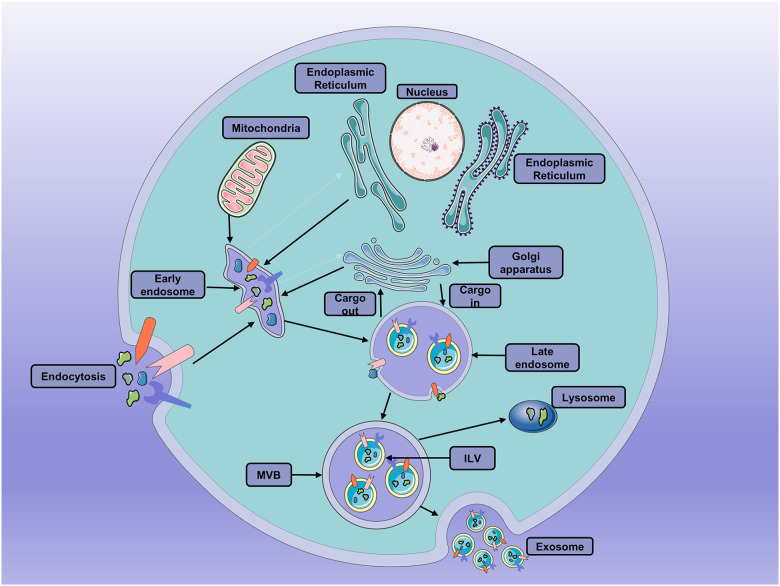
The processes of exosomes formation, secretion and fusion. Early invagination of the endosome membrane allows intracellular components to be engulfed in vesicular intraluminal vesicles (ILVs). Then, the late endosomes become multivesicular bodies (MVBs) after the selective integration of substances by early endosomes. MVBs can either fuse with the plasma membrane and then release ILVs as exosomes into the extracellular space or fuse with lysosomes or autophagosomes to be degraded.

Exosomes are small extracellular vesicles (EVs) of 40–150 nm, endosome-derived, secreted by most cells. They have been isolated from many biological fluids, including blood, urine, semen, and cerebrospinal fluid (Kalra et al., 2016). During the formation of exosomes, the first phase is the early invagination of the endosome membrane allows intracellular components to be engulfed in early sorting endosomes (ESE), with the participation of mitochondria, Golgi apparatus, and the endoplasmic reticulum (Hessvik and Llorente, 2018). Then, the ESEs can mature into the late sorting endosomes (LSEs) with the participation of the endosomal-sorting complex necessary for transport (ESCRT) proteins (Vietri et al., 2020). Eventually, the LSEs generate multivesicular bodies (MVBs) after the selective integration of substances. The MVBs contain several vesicular intraluminal vesicles (ILVs; van Niel et al., 2018). Then, MVBs fuse with the plasma membrane and then release ILVs as exosomes into the extracellular space. Also, they can fuse with lysosomes or autophagosomes to be degraded (Thery et al., 2002; Colombo et al., 2014; van Niel et al., 2018; Jeppesen et al., 2019; Figure 1) Moreover, exosomes can be taken up by recipient cells through phagocytosis, direct fusion, endocytosis, and ligand-receptor interactions. Then, the contents of the exosomes can be deposited into the cytoplasm (Kalluri and LeBleu, 2020).

**Figure 2:**
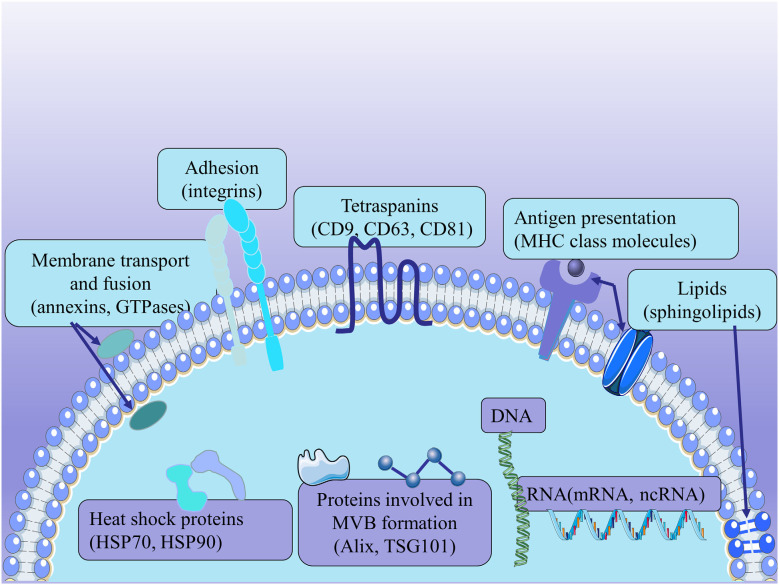
The structure of exosomes. Exosomes express the proteins involved in MVB formation (Alix and TSG101), membrane transport and fusion (annexins, GTPases), adhesion (integrins), tetraspanins (CD9, CD63, CD81), antigen presentation [major histocompatibility complex (MHC) class molecules], and heat shock proteins (HSP70, HSP90). Exosomes derived from MSCs carry a complex cargo, including nucleic acids, proteins, lipids, and enzymes.

Because exosomes are derived from endosomes, these substances, including the proteins involved in MVB formation (Alix and TSG101), membrane transport and fusion (annexins, GTPases), adhesion (integrins), tetraspanins (CD9, CD63, CD81), antigen presentation [major histocompatibility complex (MHC) class molecules], heat shock proteins (HSP70, HSP90), and other related proteins, are commonly used to identify exosomes regardless of the cell type of origin. In addition to proteins, exosomes are rich in specific lipids, mainly containing ceramide, cholesterol, and sphingolipids. And the lipids contribute to the formation and structural stability of exosomes (Mashouri et al., 2019). Exosomes also contain surface polysaccharides and glycans, mainly containing mannose, α-2,6-sialic acid, and polyglactin. They present at the plasma membrane of exosomes, contributing to the docking and attachment of these exosomes to recipient cells, especially the Glypican 1 (Melo et al., 2015). Exosomes have been reported to carry DNA and RNAs, including mRNAs and some ncRNAs (Lotvall et al., 2014; Kalra et al., 2016; Thery et al., 2018; Figure 2) Although the ability of exosomes to contain DNA remains controversial, there have been many pieces of research showing that they are used for identification (Thakur et al., 2014; Hagey et al., 2021). Exosomal RNAs are secreted to regulate intercellular communication; and miRNAs, in particular, play a vital role in various biological mechanisms (Treiber et al., 2019). However, the composition and function of exosomes remain to be fully elucidated.

**Figure 3:**
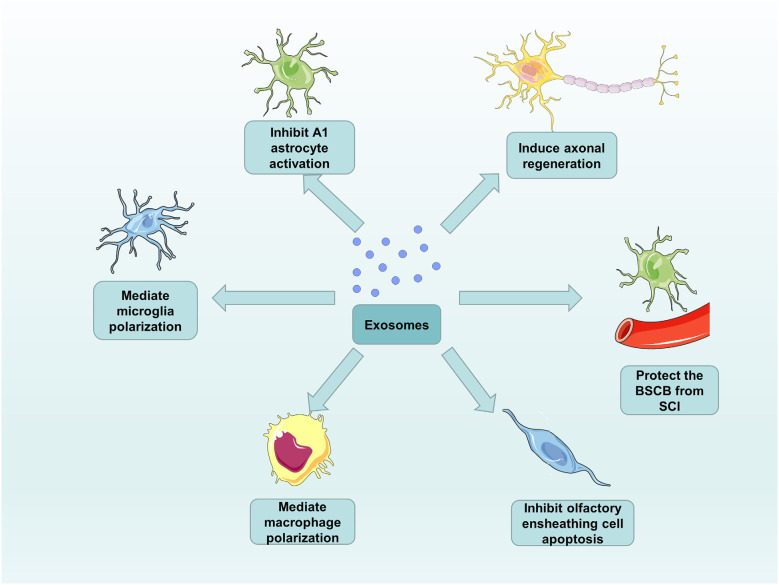
The functions of exosomes in SCI repair. Exosomes from different cell sources can inhibit A1 astrocyte activation and olfactory ensheathing cell apoptosis, as well as induce axonal regeneration, mediate microglia and macrophage polarization, and protect the BSCB from SCI.

Around 98% of all the genomic output is ncRNAs in the data from genome-wide transcriptional analysis in humans (Mattick, 2001). According to the size of ncRNAs, ncRNAs are mainly divided into two groups: long ncRNAs with more than 200 nucleotides and small ncRNAs with no more than 200 nucleotides (Mercer et al., 2009). Interestingly, although ncRNAs do not code for proteins, they have diverse functions in physiology and development of the organisms (Amaral and Mattick, 2008). For this reason, an increasing number of studies have shown that many ncRNAs, particularly noncoding small RNAs (microRNAs), long ncRNAs (lncRNAs), and circular RNAs (circRNAs) become differentially expressed after SCI. In the treatment of SCI, these ncRNAs regulate the translation and transcription mainly by targeting specific mRNAs to affect neuronal survival, axonal regeneration, and glial cell phenotype (Zhou et al., 2016; Bie et al., 2021). MicroRNAs (miRNAs) are 20–24 nucleotide RNA molecules with the function of regulating the protein expression levels by affecting mRNA. Their pivotal role in SCI can be attributed to individual miRNAs that can target the translation of many mRNAs (Bhalala et al., 2013). There are two modes of action for miRNAs binding to the 3’-untranslated region (3’-UTR) of target mRNA. One is that the perfect binding of miRNAs to targets induces mRNA degradation, and the other is that imperfect binding of miRNAs to targets represses translation (Shahzad et al., 2021). The two modes of action will prevent protein accumulation by an unknown mechanism. Furthermore, lncRNAs will act as endogenous RNA to compete for miRNAs binding to regulate gene expression. lncRNAs are defined as thousands of RNA transcripts of more than 200 nucleotides in length without protein-coding potential, which has attracted much attention in various fields (Shi et al., 2018). A study showed that the effect of miR-203 and miR-101, which can down-regulate the expression of BARD1 protein, can be counteracted by a novel lncRNA (BARD1 9’L). These findings subvert our perception of the biological function of ncRNAs, from thinking that they are nonfunctional transcriptional junk to gaining insight into their involvement in the pathogenesis of various diseases (Lee, 2012). It is reported that the exosomes play a role in intercellular communication. The attachment of RNA-induced silencing complexes (RISCs) to the ESCRT components makes the ncRNAs recruit to the exosomes (Sato-Kuwabara et al., 2015) and the ncRNAs, which are one of the enriched cargo in exosomes, can be exported outside cells to target specific mRNAs. Ceramide-dependent machinery controls the release of exosomal ncRNAs (Kosaka et al., 2010). For this reason, an increasing number of studies are focusing on the role of the exosomal ncRNAs as potential therapeutic strategies in SCI (Pant et al., 2021). Exosomes derived from different cells exhibit their functions, both *in vivo* or *in vitro*, and in healthy or disease states (Lotvall et al., 2014; Thery et al., 2018). Their differential expression in diverse states can be used as a particular biomarker, provide new therapeutic targets for diseases, and even used as a therapeutic approach to replace or assist cell therapy (Chen et al., 2017). Therefore, we summarize the respective roles of exosomes and exosomal ncRNAs obtained from different cell sources in SCI treatment with great confidence for their promotion of functional recovery after SCI (Figure 3).

### MSCs Sources

In the treatment of SCI, the beneficial effects of MSC transplants have been demonstrated in different experimental studies. There are many sources of MSCs, such as bone marrow-derived MSCs, adipose-derived MSCs, and umbilical cord-derived MSCs. Among them, bone marrow-derived MSCs are widely studied as well as MSC-derived exosomes (Liau et al., 2020; Ren et al., 2020; Andrzejewska et al., 2021). It has been reported that BMSCs-Exos effectively promotes the formation of capillaries and improves the migration of human umbilical vein endothelial cells (HUVECs) *in vitro*. In the GLU-induced excitotoxicity model, the number of TUNEL-positive neuronal cells was significantly reduced after the treatment of BMSCs-Exos, indicating that BMSCs-Exos have neuroprotective function. In an SCI rat model intravenously injected with BMSCs-Exos, the lesion area became smaller, and CSPG deposition was significantly reduced, indicating that it inhibited glial scar formation. The expression levels of inflammatory markers including TNF-α, IL-1β, and IL-6 significantly decreased, indicating the alleviation of the inflammatory response. The degree of NF200 staining reduction at the injury site was significantly lower than that in the untreated group, indicating that it promoted axonal regeneration and neuronal survival. The number of C3-positive reactive astrocytes was significantly reduced, indicating that BMSCs-Exos inhibited the activation of A1 neurotoxic reactive astrocytes (Liu et al., 2019). Moreover, some studies have shown that BMSCs-Exos effectively inhibited pericyte apoptosis and maintained BSCB integrity by regulating NOD1-related signaling pathways *in vitro*. Therefore, the pericyte level is increased, thereby enhancing the functional recovery after SCI (Zhou et al., 2022). After the treatment of BMSCs-Exos, the expression levels of autophagy-related proteins LC3B and Beclin-1 were increased. Also, the formation of autophagosomes was promoted. Besides, the expression level of caspase-3 cleaved by proapoptotic proteins was significantly decreased, while the expression of antiapoptotic protein Bcl-2 was upregulated. These results show that BMSCs-Exos can reduce neuronal apoptosis and promote the recovery of functional behavior in SCI rats by promoting autophagy, thus providing a new target for the treatment of SCI (Gu et al., 2020). Studies have shown that the complementary levels such as C6, C4 binding protein α, and complement factor H increase after SCI. Otherwise, BMSCs-Exos treatment can effectively attenuate the increasing trend of complementary levels. BMSCs-Exos can also bind to microglia at the injury site and inhibit the nuclear factor kappa-B (NF-κB) activated by SCI, thus exerting a protective effect (Zhao et al., 2019).

Studies have shown that the intravenous injection of human placental stem cell-derived exosomes (hpMSCs-Exos) significantly increased the expression of neural stem/progenitor cell markers in the spinal cord. The proliferation ability of nerve progenitor cells (NPCs) also increases, indicating that hpMSCs-Exos can promote endogenous NPC and neurogenesis activation and promote the recovery of motor and autonomic function after SCI (Zhou et al., 2021). Essentially, hpMSCs-Exos promoted vessel formation and migration of HUVECs *in vitro*, but also significantly increased the vessel number, vessel volume fraction, and vascular connectivity in a rat model of SCI (Zhang et al., 2020). The experimental results of other studies have shown that human Wharton’s jelly stem cell-derived extracellular vesicles (WJMSCs-EVs) can inhibit neuroinflammation after SCI, reduce cell death, and thus restore the motor function. WJMSCs-EVs can also decrease the GFAP expression, prevent glial scar formation, and promote regeneration by stimulating NPC (Noori et al., 2021). In *in vivo* and *in vitro* experiments, human umbilical cord stem cell-derived exosomes (hucMSCs-Exos) inhibited the secretion of their proinflammatory factors and promoted the production of anti-inflammatory factors by promoting the polarization of M1 macrophages to M2 macrophages. Ultimately, hucMSCs-Exos plays a role in controlling the inflammatory response (Sun et al., 2018). Recently, it has been shown that epidural adipose-derived exosomes (EF-MSCs-Exos) injected into the tail vein of SCI rats can improve their neurological recovery and reduce the lesion volume in SCI rats. Besides, EF-MSCs-Exos can inhibit the activation of NLRP3 inflammasome and reduce the expression of proinflammatory factors. In addition, EF-MSCs-Exos treatment can also reduce the expression level of proapoptotic protein Bax and upregulate the expression level of antiapoptotic protein Bcl-2 (Huang et al., 2020a).

Recently, it has been demonstrated that miRNAs discovery in exosomes can be exported outside cells and affect gene expression in distant cells (Colombo et al., 2014). Therefore, exosomes derived from miRNA mimics or antisense miRNAs-modified cells can overexpress or inhibit miRNAs in exosomes, thereby being used to treat SCI (Bhalala et al., 2013). Because exosomes and exosomal miRNAs from MSCs are the most widely studied to date, we summarize the existing studies on the use of MSC-derived exosomes and exosomal miRNAs for the treatment of SCI as follows (Table 1). Recent studies have also shown that the expression of lncRNAs changes after SCI, and lncRNAs might play a crucial role in the pathological process of SCI, unlike miRNAs, and lncRNAs have their specific characteristics (Yu et al., 2015). Recently, a study showed that lncRNA-Gm37494 expression was upregulated in exosomes (Exos) produced by adipose-derived stem cells (ADSCs) under hypoxia. After overexpressing in ADSCs-Exos by transfection with lncRNA-Gm37494, lncRNA-Gm37494 inhibited BV2 microglia polarization to M1 type and promote their polarization to M2 type by inhibiting miR-130b-3p and promoting PPARγ expression, ultimately achieving the purpose of repairing SCI (Shao et al., 2020).

**Table 1:** Studies about MSC-derived exosomes and exosomal miRNAs in the treatment of SCI.

**Study**	**Animal**	**Exosomal miRNAs**	**Exosomes source**	**The route of administration**	**Mechanism of action**	**Biological function**
Zhang et al. (2021)	SD rat	MiR-181c	BMSC	Tail vein injection	Upregulation of miR-181c inhibits the target gene PTEN which in turn inhibits the NF-κB signaling pathway and decreases the expression of microglial pro-inflammatory cytokines (TNF-α and IL-1β)	Reduce apoptosis and inflammation
Zhang et al. (2021)	SD rat	MiR-338–5p	BMSC	Tail vein and intrathecal injection	Upregulation of miR-338–5p represses target gene Cnr1 to regulate active Rap1 expression, activates PI3K/Akt pathway, attenuates Bax and caspase-3 expression, and up-regulates Bcl-2 expression	Reduce apoptosis and promote neuronal survival
Xiao et al. (2021)	SD rat	MiR-29b-3p	HucMSC	Tail vein injection	Upregulation of miR-29b-3p inhibits the PTEN axis while activating the Akt/mTOR pathway	Promote autophagy and axonal regeneration
Wang et al. (2021)	SD rat	MiR-199a-3p/145–5p	HucMSC	Tail vein injection	Upregulation of miR-199a-3p and miR-145–5p inhibit Cblb and Cbl, respectively, which in turn activate Akt and Erk in NGF/TrkA downstream pathways	Promote neuronal differentiation, reduce injury, and promote functional recovery
Jia et al. (2021)	SD rat	MiR-381	BMSC	Tail vein injection	Upregulation of miR-381 inhibits the BRD4-WNT5A axis while inhibiting RhoA/Rho-kinase activity	Reduce apoptosis in dorsal root ganglia (DRG)
Chen et al. (2021)	SD rat	MiR-26a	BMSC	Tail vein injection	Upregulation of miR-26a inhibits the PTEN axis while activating the Akt/mTOR pathway	Reduce glial scar formation and promote axonal regeneration
Chang et al. (2021)	SD rat	MiR-125a	BMSC	Intrathecal injection	Upregulation of miR-125a inhibits the expression of target gene IRF5	Inhibition of macrophage polarization to M1 type and secretion of proinflammatory cytokines
Liu et al. (2020)	Mice	MiR-216a-5p	BMSC	Tail vein injection	Upregulation of miR-216a-5p inhibits TLR4/NF-κB and activates the PI3K/AKT signaling pathway	Promote the microglial transition from M1 to M2 type
Li et al. (2020)	SD rat	MiR-124–3p	BMSC	Tail vein injection	Upregulation of miR-124–3p inhibits the expression of the target gene Ern1	Promote macrophage polarization to M2 type
Li et al. (2020)	SD rat	MiR-544	BMSC	Tail vein injection	Upregulation of miR-544 suppresses the expression of proinflammatory cytokines (IL-1a, TNF-a, IL-17B, and IL-36b)	Promote neuronal survival and suppresses inflammatory responses
Huang et al. (2020b)	SD rat	MiR-126	BMSC	Tail vein injection	Upregulation of miR-126 inhibits the expression of SPRED1 and PIK3R2	Promote angiogenesis and neurogenesis and reduces apoptosis
Zhou et al. (2019)	Wistar rat	MiR-21–5p	BMSC	Tail vein injection	Upregulation of miR-21–5p inhibits the expression of target gene Fasl	Reduce apoptosis
Yu et al. (2019)	SD rat	MiR-29b	BMSC	Tail vein injection	Upregulation of miR-29b promotes the expression of NF200, GAP-43, and inhibits the expression of GFAP	Promote neuronal regeneration and reduce injury
Kang et al. (2019)	SD rat	MiR-21	BMSC	Tail vein injection	Upregulation of miR-21 inhibits the expression of PTEN and PDCD4	Inhibition of cell death
Li et al. (2018)	SD rat	MiR-133b	BMSC	Tail vein injection	Upregulation of miR-133b inhibits the expression of the target gene RhoA and promotes the expression of ERK1/2, CREB, and STAT3	Inhibit neuronal cell death and enhance axonal regeneration
Ren et al. (2019)	SD rat	MiR-133b	ADSC	-	Upregulation of miR-133b inhibits the expression of the target gene RhoA and promotes the expression of CREB, STAT3, NF, GAP-43, GFAP, and MBP	Inhibit neuronal cell death and enhance axonal regeneration and neuronal survival

### NSCs/NPCs Sources

NSCs/NPCs exhibit nerve regeneration and neuroprotective effects; the transplantation of such cells into damaged tissue sites is a promising SCI therapy. Similarly, a large number of preclinical studies and some clinical studies have shown its unique advantages (Csobonyeiova et al., 2019). Nonetheless, NSCs/NPCs also suffer from the same problems as others, such as tumorigenicity and immune rejection. Fortunately, the neural stem/progenitor cell-derived exosomes (NSCs/NPCs-Exos) discovered in recent years can overcome these disadvantages of stem cell transplantation and might play the same role to a certain extent. At present, NSCs/NPCs-Exos have been gradually studied in nerve-related diseases such as stroke and brain injury, but they still need to be developed for SCI treatment (Vogel et al., 2018). Only some studies have evaluated NSCs/NPCs-Exos for the treatment of SCI. It has been demonstrated that NSCs-Exos can significantly reduce the extent of SCI and promote functional recovery and microglial activation in rats. More importantly, NSCs-Exos treatments increased the expression levels of autophagy-related proteins LC3B and Beclin-1. Also, they could promote the formation of autophagosomes. Furthermore, the expression level of caspase-3 cleaved by proapoptotic proteins clearly decreased, while the expression of antiapoptotic protein Bcl-2 was upregulated. These results indicate that NSCs-Exos reduced neuronal apoptosis and benefited the recovery of functional behavior in SCI rats by improving autophagy (Rong et al., 2019). It has also been shown that NSCs-Exos can promote the migration, proliferation, and angiogenesis of spinal cord microvascular endothelial cells (SCMECs) after trauma *in vitro*. SCMECs can also increase the microvessel density, spinal canal shrinkage, and motor function recovery in the rat models of SCI. Moreover, this study further showed that NSCs-Exos exhibited proangiogenic effects on SCMECs by transferring vascular endothelial growth factor A (VEGF-A) and enhancing microvascular regeneration and tissue healing (Zhong et al., 2020). Recently, a study showed that miR-29b expression was upregulated in exosomes (Exos) produced by human neuroepithelial stem cells (HNESCs). After overexpressing in HNESCs-Exos by transfection with miR-29b, miR-29b subsequently suppressed the apoptosis of neuron cells by down-regulating the expression of PTEN/caspase-3, ultimately achieving the purpose of repairing SCI (Kang et al., 2020). Furthermore, another study showed that miR-219a-2–3p expression was upregulated in exosomes (Exos) produced by neural stem cells (NSCs). After overexpressing in NSCs-Exos by transfection with miR-219a-2–3p, miR-219a-2–3p attenuated apoptosis and neuroinflammation by down-regulating the expression of YY1/NF-κB, ultimately achieving the purpose of repairing SCI (Ma et al., 2019).

### Other Cell Sources

Nerve regeneration is related to various cells in the tissue microenvironment, and different types of cells in diverse states produce the corresponding effects on the target recipient cells. For example, SCs cannot only migrate to the damaged tissue area to become a key component of nerve regeneration but also secrete signaling molecules to attract macrophages, activate local MSC, and interact with other cell types (Min et al., 2021). SC-derived exosomes obtained from the skin (SKP-SCs-Exos) have also been studied; they can regulate cell growth and death signaling pathways mediated by Akt/mTOR/p70S6K. SKP-SCs-Exos can enhance the recovery of neuronal viability and axonal regeneration in the *in vivo* and *in vitro* models (Wu et al., 2020). It has also been shown that SC-derived exosomes (SCDEs) obtained from the sciatic nerve can reduce the deposition of chondroitin sulfate proteoglycans (CSPGs) deposition by increasing Toll-like receptor 2 (TLR2) expression on astrocytes through the NF-κB/PI3K signaling pathway, thereby promoting the functional recovery in mice after SCI (Pan et al., 2021). Recently, it has been demonstrated that peripheral macrophages (PMs) can effectively improve the microenvironment of the lesion site, and they are a pivotal factor in promoting the repair after SCI (Tsarouchas et al., 2018). Then, the mechanism of peripheral macrophage-derived exosomes (PMs-Exos) in the treatment of SCI has also been elucidated. PMs-Exos can activate microglial autophagy and enhance the polarization of anti-inflammatory microglia (M2) by inhibiting the PI3K/AKT/mTOR signaling pathway, thus playing a meaningful role in the anti-inflammation process of SCI repair (Zhang B. et al., 2021). Transplantation of OECs also has its unique advantages in SCI treatment, and it has a strong growth force. Particularly, it provides a suitable microenvironment and strong migration characteristics for axonal growth (Kato et al., 2000). HOECs-Exos can stimulate NPC proliferation to promote nerve regeneration in the *in vitro* models and improve NPC cytotoxicity during oxidative stress. Otherwise, the *in vivo* therapeutic effect in SCI rat model remains to be studied (Tu and Hsueh, 2020). Pericytes, a vital part of the neurovascular unit, have the same characteristics as stem cells. Moreover, pericytes interact with endothelial cells and maintain the stability of endothelial barrier. Treatment with pericyte-derived exosomes can promote blood flow and endothelial function to protect BSCB. Moreover, pericyte-derived exosomes can improve the functional and behavioral recovery after SCI by reducing the apoptotic response, and they can be cocultured with endothelial cells under hypoxic conditions *in vitro* can also reduce their permeability and play a protective role (Yuan et al., 2019). Recently, a study showed that miR-421-3p expression was upregulated in exosomes (Exos) produced by M2 bone marrow-derived macrophages (BMDMs). After overexpressing in BMDMs-Exos by transfection with miR-421-3p, miR-421-3p enhanced protective autophagy in neuronal cells by inhibiting the expression of mTOR protein, ultimately achieving the purpose of repairing SCI (Wang et al., 2020).

## The Separation and Concentration Methods of Each Cell-Derived Exosomes in The Treatment of SCI

MSCs-Exos were separated and concentrated by ultracentrifugation and differential centrifugation in many studies (Huang et al., 2017, 2020a; Lankford et al., 2018; Wang et al., 2018; Guo et al., 2019; Ji et al., 2019; Kang et al., 2019; Li et al., 2019; Li C. et al., 2020; Li et al., 2021; Chen et al., 2021; Cheng et al., 2021; Han et al., 2021; Jia et al., 2021; Jiang and Zhang, 2021; Liu W. Z. et al., 2021; Liu et al., 2022; Nakazaki et al., 2021; Nie and Jiang, 2021; Noori et al., 2021; Sheng et al., 2021; Xiao et al., 2021; Xin et al., 2021; Zhang A. et al., 2021; Liang et al., 2022; Zhou et al., 2022) and by precipitation kits/polymer (PEG or others) in some studies (Li et al., 2018; Ren et al., 2019; Xu et al., 2019; Yu et al., 2019; Zhao et al., 2019; Fan et al., 2021; Jia et al., 2021a, b; Kang and Guo, 2022). To achieve better specificity of MSCs-Exos separation, many researchers isolated MSCs-Exos using ultrafiltration-centrifugation combined with density-gradient ultracentrifugation (Liu et al., 2020; Shao et al., 2020; Chang et al., 2021; Luo et al., 2021; Huang et al., 2022). Furthermore, some researchers used one or more techniques following the ultracentrifugation, such as density-gradient ultracentrifugation (Liu et al., 2019), size-exclusion chromatography (Li L. et al., 2020), and magnetic sorting (Kim et al., 2018). NSCs-Exos were separated and concentrated by ultracentrifugation (Ma et al., 2019), ultrafiltration-centrifugation (Zhong et al., 2020), and by kits methods (Kang et al., 2020). To achieve better specificity of NSCs-Exos separation, some researchers isolated NSCs-Exos using ultrafiltration-centrifugation combined with density-gradient ultracentrifugation (Rong et al., 2019). SKP-SCs-Exos were separated and concentrated by kits methods (Wu et al., 2020). SC-Exos (Pan et al., 2021), HOECs-Exos (Tu and Hsueh, 2020), and PMs-Exos (Zhang B. et al., 2021) were separated and concentrated by ultracentrifugation. BMDMs-Exos were separated and concentrated by ultracentrifugation and kits methods (Wang et al., 2020). There are no specific separation and concentration methods for exosomes from different cell sources. However, as reviewed in the 2018 guidelines, different isolation methods have their advantages and disadvantages (Thery et al., 2018). For example, the ultracentrifugation method has intermediate recovery and intermediate specificity. The kit method has high recovery but low specificity. Moreover, ultrafiltration-centrifugation combined with density-gradient ultracentrifugation has low recovery but high specificity. Although there are no high recovery and high specificity exosome isolation methods, dendritic cells (DCs)-derived exosomes have been applied in clinical trials to treat patients with malignant melanoma and non-small cell lung carcinoma and achieved some efficacy (Nikfarjam et al., 2020).

## Prospects

Traditional drugs have numerous disadvantages: poor water solubility, rapid *in vivo* clearance, poor biocompatibility, unsatisfactory *in vivo* distribution, and low permeability. New drug carriers are being continuously developed to optimize and improve the bioavailability to solve these problems. In recent years, exosomes have also been developed for drug loading, which can improve the stability of drugs and exhibit natural targeting ability based on donor cells. Because it is a nanomolecule with cell surface substances, it can readily and selectively penetrate biological barriers (Batrakova and Kim, 2015; Antimisiaris et al., 2018). At present, there are two main methods of exosomes transplantation in the treatment of SCI (Table 1), including intrathecal injection and tail vein injection. Moreover, a meta-analysis showed that intrathecal therapy seems to be more effective than tail vein injection therapy (Yi and Wang, 2021). Interestingly, a study showed that exosomes could also be used to treat SCI by intranasal injection. More specifically, intranasal exosomes led to significant locomotor recovery as compared to the intrathecal exosomes group (Shahzad et al., 2021). However, so far, there is no uniform standard for the isolation methods of exosomes. Exosomes are obtained using different isolation methods with different purities and specificities. At present, the most used isolation method for exosomes is ultracentrifugation, but the purity of obtained exosomes is low. Therefore, the isolation methods of exosomes still should be developed to obtain a higher purity and specificity. For questions regarding exosome preservation and transport, refer to the 2018 International Association for Extracellular Vesicles statement (Gardiner et al., 2016; Thery et al., 2018). When exosomes are used as drugs or carriers for treatments, the dosage, timing, and administration route are still not known. Therefore, it is essential to assess their half-life and *in vivo* distribution characteristics in advance (Smyth et al., 2015; Yi and Wang, 2021). Regarding the content and function of exosomes, as mentioned earlier, the results obtained from different cell sources and culture conditions are different, and the diversity in their therapeutic effects remains to be studied. Specific exosomes can be selected according to their studies. Moreover, more exosomes obtained from cell sources and culture conditions can be sought.

Currently, in the field of treatment of SCI, most studies on exosomal ncRNAs focused on miRNAs, lncRNAs, and circRNAs. In the future, more RNAs should be developed, such as rRNA, tRNA, and piRNA (Chandran et al., 2017; Jogia and Ruitenberg, 2020). For the targeted therapy of ncRNAs, the biological regulatory pathway is very complex, and a single targeted axis might play only a limited role. Therefore, to clarify the regulatory network, multicellular, multitarget, and multipathway validation is one of the directions to elucidate the specific regulatory mechanism for future research (Li X. et al., 2020; Wang W. Z. et al., 2021). Nevertheless, ncRNA-targeted therapy has many disadvantages. For example, it is easy to miss the target. Even it has a low transfection efficiency and a short half-life. What’s worse, it is difficult to overcome the limitations of blood-spinal cord barrier (Shahzad et al., 2021). Fortunately, targeting and half-life can be improved by the carrier delivery of drugs such as viruses, siRNAs, lipids, polyethylene glycol, and exosomes (Guo et al., 2019; Xu et al., 2019; Li L. et al., 2020; Segel et al., 2021). What’s more, as mentioned earlier, many studies have achieved some results using ncRNAs in combination with exosomes for the treatment of SCI. Because exosomes have the ability to penetrate the BSCB, they can deliver ncRNAs to the lesioned area of SCI to enhance efficacy (Ding et al., 2019). Moreover, exosomes have a lower risk of tumorigenicity, toxic effects, and autoimmune responses (Lai et al., 2013). However, the clinical application of exosomes remains problematic. It has been shown that the amount of exosomes produced by cellular secretion is small (Katsuda et al., 2013), and it is hard to meet clinical needs. Therefore, regulating exosomes release is particularly important. It is reported that the pH of the microenvironment has a role in the secretion of exosomes (Parolini et al., 2009). Regulating the pH of the microenvironment may result in increased exosomes production. Furthermore, most studies have focused on the mechanism of miRNAs in combination with exosomes for the treatment of SCI, and only a few studies have used other RNAs.

## Conclusion

In summary, the treatment of SCI is still a huge concern, and no effective methods are available to promote neurological recovery. SCI is the result of multiple factors, hindering the development of rehabilitation because of its complexity. Therefore, understanding the pathomechanism will facilitate better treatment of SCI. As a mediator of intercellular communication, exosomes are advantageous in treating SCI. Exosomal ncRNAs have also been shown to contribute to nerve regeneration. Exosomes derived from different cells play very much the same role, and exosomal ncRNAs such as miRNAs, lncRNAs, and circRNAs have tremendous therapeutic potential in SCI. We must optimize and enrich exosomes and exosomal ncRNAs of various cellular sources and combine both of them effectively to improve their therapeutic efficacy in SCI. Then, more studies are needed to elucidate the specific mechanism of action of exosomes and exosomal ncRNAs from different cell sources in SCI. These searches provide a comprehensive theoretical basis for the clinical translation of exosomes and exosomal ncRNAs from different cell sources in SCI treatment and provide great hope for the clinical treatment of SCI.

## Author Contributions

All the listed authors directly, substantially, and intellectually contributed to the preparation of this manuscript and approved the publication. All authors contributed to the article and approved the submitted version.

## Conflict of Interest

The authors declare that the research was conducted in the absence of any commercial or financial relationships that could be construed as a potential conflict of interest.

## Publisher’s Note

All claims expressed in this article are solely those of the authors and do not necessarily represent those of their affiliated organizations, or those of the publisher, the editors and the reviewers. Any product that may be evaluated in this article, or claim that may be made by its manufacturer, is not guaranteed or endorsed by the publisher.

## Abbreviations

SCI: spinal cord injury
ECM: extracellular matrix
ncRNAs: noncoding RNAs
iPSC: induced pluripotent stem cells
MSCs: mesenchymal stem cells
NSCs: neural stem cells
OPCs: oligodendrocyte progenitor cells
SCs: schwann cells
OECs: olfactory ensheathing cells
BSCB: blood-spinal cord barrier
CSPGs: chondroitin sulfate proteoglycans
PTPσ: Protein Tyrosine Phosphatase Sigma
EVs: extracellular vesicles
ESE: early sorting endosomes
LSEs: late sorting endosomes
ESCRT: endosomal-sorting complex necessary for transport
MVBs: multivesicular bodies
ILVs: intraluminal vesicles
MHC: major histocompatibility complex
HSP: heat shock proteins
HUVECs: human umbilical vein endothelial cells
BMSCs-Exos: bone marrow mesenchymal stem cells-derived exosomes
NF-κB: nuclear factor kappa-B
hpMSCs-Exos: human placental stem cell-derived exosomes
NPCs: nerve progenitor cells
WJMSCs-EVs: Wharton’s jelly stem cell-derived extracellular vesicles
hucMSCs-Exos: human umbilical cord stem cell-derived exosomes
EF-MSCs-Exos: epidural adipose-derived exosomes
NSCs/NPCs-Exos: neural stem/progenitor cell-derived exosomes
SCMECs: spinal cord microvascular endothelial cells
VEGF-A: vascular endothelial growth factor A
SKP-SCs-Exos: skin schwann cells-derived exosomes
SCDEs: SC-derived exosomes
TLR2: toll-like receptor 2
PMs: peripheral macrophages
PMs-Exos: peripheral macrophage-derived exosomes
noncoding small RNAs: microRNAs
lncRNAs: long ncRNAs
circRNAs: circular RNAs
miRNAs: microRNAs
PTENP1: phosphatase and tension protein homologous pseudogene 1
PTEN: phosphatase and tension protein homolog
ADSCs: adipose-derived stem cells
Exos: exosomes
BMDMs: bone marrow-derived macrophages
HNESCs: human neuroepithelial stem cells
DCs: dendritic cells.

## References

[B1] Ahuja C. S. Mothe A. Khazaei M. Badhiwala J. H. Gilbert E. A. van der Kooy D.. (2020). The leading edge: emerging neuroprotective and neuroregenerative cell-based therapies for spinal cord injury. Stem Cells Transl. Med. 9, 1509–1530. 10.1002/sctm.19-0135 32691994 PMC7695641

[B2] Ahuja C. S. Wilson J. R. Nori S. Kotter M. R. N. Druschel C. Curt A.. (2017). Traumatic spinal cord injury. Nat. Rev. Dis. Primers 3:17018. 10.1038/nrdp.2017.18 28447605

[B3] Amaral P. P. Mattick J. S. (2008). Noncoding RNA in development. Mamm. Genome 19, 454–492. 10.1007/s00335-008-9136-7 18839252

[B4] Andrzejewska A. Dabrowska S. Lukomska B. Janowski M. (2021). Mesenchymal stem cells for neurological disorders. Adv. Sci. (Weinh) 8:2002944. 10.1002/advs.202002944 33854883 PMC8024997

[B5] Antimisiaris S. G. Mourtas S. Marazioti A. (2018). Exosomes and exosome-inspired vesicles for targeted drug delivery. Pharmaceutics 10:218. 10.3390/pharmaceutics10040218 30404188 PMC6321407

[B6] Batrakova E. V. Kim M. S. (2015). Using exosomes, naturally-equipped nanocarriers, for drug delivery. J. Control. Release 219, 396–405. 10.1016/j.jconrel.2015.07.030 26241750 PMC4656109

[B7] Bhalala O. G. Srikanth M. Kessler J. A. (2013). The emerging roles of microRNAs in CNS injuries. Nat. Rev. Neurol. 9, 328–339. 10.1038/nrneurol.2013.67 23588363 PMC3755895

[B8] Bie F. Wang K. Xu T. Yuan J. Ding H. Lv B.. (2021). The potential roles of circular RNAs as modulators in traumatic spinal cord injury. Biomed. Pharmacother. 141:111826. 10.1016/j.biopha.2021.111826 34328121

[B9] Bradbury E. J. Burnside E. R. (2019). Moving beyond the glial scar for spinal cord repair. Nat. Commun. 10:3879. 10.1038/s41467-019-11707-7 31462640 PMC6713740

[B10] Chandran R. Mehta S. L. Vemuganti R. (2017). Non-coding RNAs and neuroprotection after acute CNS injuries. Neurochem. Int. 111, 12–22. 10.1016/j.neuint.2017.01.015 28131900 PMC5529259

[B11] Chang Q. Hao Y. Wang Y. Zhou Y. Zhuo H. Zhao G. (2021). Bone marrow mesenchymal stem cell-derived exosomal microRNA-125a promotes M2 macrophage polarization in spinal cord injury by downregulating IRF5. Brain Res. Bull. 170, 199–210. 10.1016/j.brainresbull.2021.02.015 33609602

[B12] Chen B. Li Q. Zhao B. Wang Y. (2017). Stem cell-derived extracellular vesicles as a novel potential therapeutic tool for tissue repair. Stem Cells Transl. Med. 6, 1753–1758. 10.1002/sctm.16-0477 28653443 PMC5689748

[B15] Chen Y. Tian Z. He L. Liu C. Wang N. Rong L.. (2021). Exosomes derived from miR-26a-modified MSCs promote axonal regeneration *via* the PTEN/AKT/mTOR pathway following spinal cord injury. Stem Cell Res. Ther. 12:224. 10.1186/s13287-021-02282-0 33820561 PMC8022427

[B16] Cheng J. Chen Z. Liu C. Zhong M. Wang S. Sun Y.. (2021). Bone mesenchymal stem cell-derived exosome-loaded injectable hydrogel for minimally invasive treatment of spinal cord injury. Nanomedicine (Lond) 16, 1567–1579. 10.2217/nnm-2021-0025 34189939

[B17] Cho K. H. T. Xu B. Blenkiron C. Fraser M. (2019). Emerging roles of miRNAs in brain development and perinatal brain injury. Front. Physiol. 10:227. 10.3389/fphys.2019.00227 30984006 PMC6447777

[B18] Colombo M. Raposo G. Théry C. (2014). Biogenesis, secretion and intercellular interactions of exosomes and other extracellular vesicles. Annu. Rev. Cell Dev. Biol. 30, 255–289. 10.1146/annurev-cellbio-101512-122326 25288114

[B19] Csobonyeiova M. Polak S. Zamborsky R. Danisovic L. (2019). Recent progress in the regeneration of spinal cord injuries by induced pluripotent stem cells. Int. J. Mol. Sci. 20:3838. 10.3390/ijms20153838 31390782 PMC6695701

[B20] Ding S. Q. Chen J. Wang S. N. Duan F. X. Chen Y. Q. Shi Y. J.. (2019). Identification of serum exosomal microRNAs in acute spinal cord injured rats. Exp. Biol. Med. (Maywood) 244, 1149–1161. 10.1177/1535370219872759 31450959 PMC6802156

[B21] Donnelly D. J. Popovich P. G. (2008). Inflammation and its role in neuroprotection, axonal regeneration and functional recovery after spinal cord injury. Exp. Neurol. 209, 378–388. 10.1016/j.expneurol.2007.06.009 17662717 PMC2692462

[B22] Dutta D. Khan N. Wu J. Jay S. M. (2021). Extracellular vesicles as an emerging frontier in spinal cord injury pathobiology and therapy. Trends Neurosci. 44, 492–506. 10.1016/j.tins.2021.01.003 33581883 PMC8159852

[B23] Ehlers M. D. (2004). Deconstructing the axon: wallerian degeneration and the ubiquitin-proteasome system. Trends Neurosci. 27, 3–6. 10.1016/j.tins.2003.10.015 14698600

[B24] Fan L. Dong J. He X. Zhang C. Zhang T. (2021). Bone marrow mesenchymal stem cells-derived exosomes reduce apoptosis and inflammatory response during spinal cord injury by inhibiting the TLR4/MyD88/NF-κB signaling pathway. Hum. Exp. Toxicol. 40, 1612–1623. 10.1177/09603271211003311 33779331

[B25] Feng J. Zhang Y. Zhu Z. Gu C. Waqas A. Chen L. (2021). Emerging exosomes and exosomal MiRNAs in spinal cord injury. Front. Cell. Dev. Biol. 9:703989. 10.3389/fcell.2021.703989 34307384 PMC8299525

[B26] Gardiner C. Di Vizio D. Sahoo S. Thery C. Witwer K. W. Wauben M.. (2016). Techniques used for the isolation and characterization of extracellular vesicles: results of a worldwide survey. J. Extracell. Vesicles 5:32945. 10.3402/jev.v5.32945 27802845 PMC5090131

[B27] Gómara-Toldrà N. Sliwinski M. Dijkers M. P. (2014). Physical therapy after spinal cord injury: a systematic review of treatments focused on participation. J. Spinal Cord Med. 37, 371–379. 10.1179/2045772314Y.0000000194 24621042 PMC4116720

[B28] Gu J. Jin Z. S. Wang C. M. Yan X. F. Mao Y. Q. Chen S. (2020). Bone marrow mesenchymal stem cell-derived exosomes improves spinal cord function after injury in rats by activating autophagy. Drug Des. Devel. Ther. 14, 1621–1631. 10.2147/DDDT.S237502 32425507 PMC7196809

[B29] Guo S. Perets N. Betzer O. Ben-Shaul S. Sheinin A. Michaelevski I.. (2019). Intranasal delivery of mesenchymal stem cell derived exosomes loaded with phosphatase and tensin homolog siRNA repairs complete spinal cord injury. ACS Nano 13, 10015–10028. 10.1021/acsnano.9b01892 31454225

[B30] Hagey D. W. Kordes M. Gorgens A. Mowoe M. O. Nordin J. Z. Moro C. F.. (2021). Extracellular vesicles are the primary source of blood-borne tumour-derived mutant KRAS DNA early in pancreatic cancer. J. Extracell. Vesicles 10:e12142. 10.1002/jev2.12142 34595842 PMC8485184

[B31] Han T. Song P. Wu Z. Xiang X. Liu Y. Wang Y.. (2021). MSC secreted extracellular vesicles carrying TGF-beta upregulate Smad 6 expression and promote the regrowth of neurons in spinal cord injured rats. Stem Cell Rev. Rep. 10.1007/s12015-021-10219-6. [Online ahead of print]. 34449013 PMC8942898

[B32] Harrop J. S. Hashimoto R. Norvell D. Raich A. Aarabi B. Grossman R. G.. (2012). Evaluation of clinical experience using cell-based therapies in patients with spinal cord injury: a systematic review. J. Neurosurg. Spine 17, 230–246. 10.3171/2012.5.AOSPINE12115 22985383

[B34] Hessvik N. P. Llorente A. (2018). Current knowledge on exosome biogenesis and release. Cell. Mol. Life Sci. 75, 193–208. 10.1007/s00018-017-2595-9 28733901 PMC5756260

[B35] Huang J. H. Fu C. H. Xu Y. Yin X. M. Cao Y. Lin F. Y. (2020a). Extracellular vesicles derived from epidural fat-mesenchymal stem cells attenuate NLRP3 inflammasome activation and improve functional recovery after spinal cord injury. Neurochem. Res. 45, 760–771. 10.1007/s11064-019-02950-x 31953741

[B36] Huang J. H. Xu Y. Yin X. M. Lin F. Y. (2020b). Exosomes derived from miR-126-modified MSCs promote angiogenesis and neurogenesis and attenuate apoptosis after spinal cord injury in rats. Neuroscience 424, 133–145. 10.1016/j.neuroscience.2019.10.043 31704348

[B38] Huang T. Jia Z. Fang L. Cheng Z. Qian J. Xiong F.. (2022). Extracellular vesicle-derived miR-511-3p from hypoxia preconditioned adipose mesenchymal stem cells ameliorates spinal cord injury through the TRAF6/S1P axis. Brain Res. Bull. 180, 73–85. 10.1016/j.brainresbull.2021.12.015 34974133

[B37] Huang J. H. Yin X. M. Xu Y. Xu C. C. Lin X. Ye F. B.. (2017). Systemic administration of exosomes released from mesenchymal stromal cells attenuates apoptosis, inflammation and promotes angiogenesis after spinal cord injury in rats. J. Neurotrauma 34, 3388–3396. 10.1089/neu.2017.5063 28665182

[B39] Hurlbert R. J. Hadley M. N. Walters B. C. Aarabi B. Dhall S. S. Gelb D. E.. (2015). Pharmacological therapy for acute spinal cord injury. Neurosurgery 76, S71–S83. 10.1227/01.neu.0000462080.04196.f7 25692371

[B40] Jeppesen D. K. Fenix A. M. Franklin J. L. Higginbotham J. N. Zhang Q. Zimmerman L. J.. (2019). Reassessment of exosome composition. Cell 177, 428–445.e18. 10.1016/j.cell.2019.02.029 30951670 PMC6664447

[B41] Ji W. Jiang W. Li M. Li J. Li Z. (2019). miR-21 deficiency contributes to the impaired protective effects of obese rat mesenchymal stem cell-derived exosomes against spinal cord injury. Biochimie 167, 171–178. 10.1016/j.biochi.2019.10.002 31605737

[B42] Jia X. Huang G. Wang S. Long M. Tang X. Feng D.. (2021). Extracellular vesicles derived from mesenchymal stem cells containing microRNA-381 protect against spinal cord injury in a rat model *via* the BRD4/WNT5A axis. Bone Joint Res. 10, 328–339. 10.1302/2046-3758.105.BJR-2020-0020.R1 34024119 PMC8160032

[B43] Jia Y. Lu T. Chen Q. Pu X. Ji L. Yang J.. (2021a). Exosomes secreted from sonic hedgehog-modified bone mesenchymal stem cells facilitate the repair of rat spinal cord injuries. Acta Neurochir. (Wien) 163, 2297–2306. 10.1007/s00701-021-04829-9 33821317 PMC8270837

[B44] Jia Y. Yang J. Lu T. Pu X. Chen Q. Ji L.. (2021b). Repair of spinal cord injury in rats *via* exosomes from bone mesenchymal stem cells requires sonic hedgehog. Regen. Ther. 18, 309–315. 10.1016/j.reth.2021.08.007 34522723 PMC8416644

[B46] Jiang Z. Zhang J. (2021). Mesenchymal stem cell-derived exosomes containing miR-145-5p reduce inflammation in spinal cord injury by regulating the TLR4/NF-κB signaling pathway. Cell Cycle 20, 993–1009. 10.1080/15384101.2021.1919825 33945431 PMC8172161

[B47] Jogia T. Ruitenberg M. J. (2020). Traumatic spinal cord injury and the gut microbiota: current insights and future challenges. Front. Immunol. 11:704. 10.3389/fimmu.2020.00704 32528463 PMC7247863

[B48] Kalluri R. LeBleu V. S. (2020). The biology, function and biomedical applications of exosomes. Science 367:eaau6977. 10.1126/science.aau6977 32029601 PMC7717626

[B49] Kalra H. Drummen G. P. Mathivanan S. (2016). Focus on extracellular vesicles: introducing the next small big thing. Int. J. Mol. Sci. 17:170. 10.3390/ijms17020170 26861301 PMC4783904

[B50] Kang J. Guo Y. (2022). Human umbilical cord mesenchymal stem cells derived exosomes promote neurological function recovery in a rat spinal cord injury model. Neurochem. Res. 10.1007/s11064-022-03545-9. [Online ahead of print]. 35132478

[B51] Kang J. Li Z. Zhi Z. Wang S. Xu G. (2019). MiR-21 derived from the exosomes of MSCs regulates the death and differentiation of neurons in patients with spinal cord injury. Gene Ther. 26, 491–503. 10.1038/s41434-019-0101-8 31570818

[B52] Kang J. Zhang C. Zhi Z. Wang Y. Liu J. Wu F.. (2020). Stem-like cells of various origins showed therapeutic effect to improve the recovery of spinal cord injury. Artif. Cells Nanomed. Biotechnol. 48, 627–638. 10.1080/21691401.2020.1725031 32054316

[B53] Kato T. Honmou O. Uede T. Hashi K. Kocsis J. D. (2000). Transplantation of human olfactory ensheathing cells elicits remyelination of demyelinated rat spinal cord. Glia 30, 209–218. 10.1002/(sici)1098-1136(200005)30:3<209::aid-glia1>3.0.co;2-8 10756071 PMC2605375

[B54] Katsuda T. Tsuchiya R. Kosaka N. Yoshioka Y. Takagaki K. Oki K.. (2013). Human adipose tissue-derived mesenchymal stem cells secrete functional neprilysin-bound exosomes. Sci. Rep. 3:1197. 10.1038/srep01197 23378928 PMC3561625

[B55] Kim H. Y. Kumar H. Jo M. J. Kim J. Yoon J. K. Lee J. R.. (2018). Therapeutic efficacy-potentiated and diseased organ-targeting nanovesicles derived from mesenchymal stem cells for spinal cord injury treatment. Nano Lett. 18, 4965–4975. 10.1021/acs.nanolett.8b01816 29995418

[B56] Kosaka N. Iguchi H. Ochiya T. (2010). Circulating microRNA in body fluid: a new potential biomarker for cancer diagnosis and prognosis. Cancer Sci. 101, 2087–2092. 10.1111/j.1349-7006.2010.01650.x 20624164 PMC11159200

[B57] Kumar H. Ropper A. E. Lee S. H. Han I. (2017). Propitious therapeutic modulators to prevent blood-spinal cord barrier disruption in spinal cord injury. Mol. Neurobiol. 54, 3578–3590. 10.1007/s12035-016-9910-6 27194298

[B58] Lai R. C. Yeo R. W. Tan K. H. Lim S. K. (2013). Exosomes for drug delivery - a novel application for the mesenchymal stem cell. Biotechnol. Adv. 31, 543–551. 10.1016/j.biotechadv.2012.08.008 22959595

[B59] Lankford K. L. Arroyo E. J. Nazimek K. Bryniarski K. Askenase P. W. Kocsis J. D. (2018). Intravenously delivered mesenchymal stem cell-derived exosomes target M2-type macrophages in the injured spinal cord. PLoS One 13:e0190358. 10.1371/journal.pone.0190358 29293592 PMC5749801

[B60] Lee J. T. (2012). Epigenetic regulation by long noncoding RNAs. Science 338, 1435–1439. 10.1126/science.1231776 23239728

[B61] Lee J. Y. Kim H. S. Choi H. Y. Oh T. H. Yune T. Y. (2012). Fluoxetine inhibits matrix metalloprotease activation and prevents disruption of blood-spinal cord barrier after spinal cord injury. Brain 135, 2375–2389. 10.1093/brain/aws171 22798270

[B62] Li C. Jiao G. Wu W. Wang H. Ren S. Zhang L.. (2019). Exosomes from bone marrow mesenchymal stem cells inhibit neuronal apoptosis and promote motor function recovery *via* the Wnt/β-catenin signaling pathway. Cell Transplant. 28, 1373–1383. 10.1177/0963689719870999 31423807 PMC6802144

[B63] Li C. Li X. Zhao B. Wang C. (2020). Exosomes derived from miR-544-modified mesenchymal stem cells promote recovery after spinal cord injury. Arch. Physiol. Biochem. 126, 369–375. 10.1080/13813455.2019.1691601 32141339

[B68] Li X. Lou X. Xu S. Du J. Wu J. (2020). Hypoxia inducible factor-1 (HIF-1α) reduced inflammation in spinal cord injury *via* miR-380-3p/ NLRP3 by Circ 0001723. Biol. Res. 53:35. 10.1186/s40659-020-00302-6 32819442 PMC7439692

[B64] Li C. Qin T. Zhao J. He R. Wen H. Duan C.. (2021). Bone marrow mesenchymal stem cell-derived exosome-educated macrophages promote functional healing after spinal cord injury. Front. Cell. Neurosci. 15:725573. 10.3389/fncel.2021.725573 34650405 PMC8506031

[B66] Li L. Zhang Y. Mu J. Chen J. Zhang C. Cao H.. (2020). Transplantation of human mesenchymal stem-cell-derived exosomes immobilized in an adhesive hydrogel for effective treatment of spinal cord injury. Nano Lett. 20, 4298–4305. 10.1021/acs.nanolett.0c00929 32379461

[B65] Li D. Zhang P. Yao X. Li H. Shen H. Li X.. (2018). Exosomes derived from miR-133b-modified mesenchymal stem cells promote recovery after spinal cord injury. Front. Neurosci. 12:845. 10.3389/fnins.2018.00845 30524227 PMC6262643

[B67] Li R. Zhao K. Ruan Q. Meng C. Yin F. (2020). Bone marrow mesenchymal stem cell-derived exosomal microRNA-124-3p attenuates neurological damage in spinal cord ischemia-reperfusion injury by downregulating Ern1 and promoting M2 macrophage polarization. Arthritis Res. Ther. 22:75. 10.1186/s13075-020-2146-x 32272965 PMC7146970

[B69] Liang Y. Wu J. H. Zhu J. H. Yang H. (2022). Exosomes secreted by hypoxia-pre-conditioned adipose-derived mesenchymal stem cells reduce neuronal apoptosis in rats with spinal cord injury. J. Neurotrauma 10.1089/neu.2021.0290. [Online ahead of print]. 35018814

[B70] Liau L. L. Looi Q. H. Chia W. C. Subramaniam T. Ng M. H. Law J. X. (2020). Treatment of spinal cord injury with mesenchymal stem cells. Cell Biosci. 10:112. 10.1186/s13578-020-00475-3 32983406 PMC7510077

[B71] Liu C. Hu F. Jiao G. Guo Y. Zhou P. Zhang Y.. (2022). Dental pulp stem cell-derived exosomes suppress M1 macrophage polarization through the ROS-MAPK-NFκB P65 signaling pathway after spinal cord injury. J. Nanobiotechnology 20:65. 10.1186/s12951-022-01273-4 35109874 PMC8811988

[B72] Liu J. Lin M. Qiao F. Zhang C. (2021). Exosomes derived from lncRNA TCTN2-modified mesenchymal stem cells improve spinal cord injury by miR-329-3p/IGF1R Axis. J. Mol. Neurosci. 72, 482–495. 10.1007/s12031-021-01914-7 34623606

[B75] Liu W. Z. Ma Z. J. Li J. R. Kang X. W. (2021). Mesenchymal stem cell-derived exosomes: therapeutic opportunities and challenges for spinal cord injury. Stem Cell Res. Ther. 12:102. 10.1186/s13287-021-02153-8 33536064 PMC7860030

[B73] Liu W. Rong Y. Wang J. Zhou Z. Ge X. Ji C.. (2020). Exosome-shuttled miR-216a-5p from hypoxic preconditioned mesenchymal stem cells repair traumatic spinal cord injury by shifting microglial M1/M2 polarization. J. Neuroinflammation 17:47. 10.1186/s12974-020-1726-7 32019561 PMC7001326

[B74] Liu W. Wang Y. Gong F. Rong Y. Luo Y. Tang P.. (2019). Exosomes derived from bone mesenchymal stem cells repair traumatic spinal cord injury by suppressing the activation of A1 neurotoxic reactive astrocytes. J. Neurotrauma 36, 469–484. 10.1089/neu.2018.5835 29848167

[B76] Lotvall J. Hill A. F. Hochberg F. Buzas E. I. Di Vizio D. Gardiner C.. (2014). Minimal experimental requirements for definition of extracellular vesicles and their functions: a position statement from the International Society for Extracellular Vesicles. J. Extracell. Vesicles 3:26913. 10.3402/jev.v3.26913 25536934 PMC4275645

[B77] Lukovic D. Stojkovic M. Moreno-Manzano V. Jendelova P. Sykova E. Bhattacharya S. S.. (2015). Concise review: reactive astrocytes and stem cells in spinal cord injury: good guys or bad guys? Stem Cells 33, 1036–1041. 10.1002/stem.1959 25728093

[B78] Luo Y. Xu T. Liu W. Rong Y. Wang J. Fan J.. (2021). Exosomes derived from GIT1-overexpressing bone marrow mesenchymal stem cells promote traumatic spinal cord injury recovery in a rat model. Int. J. Neurosci. 131, 170–182. 10.1080/00207454.2020.1734598 32223487

[B79] Ma K. Xu H. Zhang J. Zhao F. Liang H. Sun H.. (2019). Insulin-like growth factor-1 enhances neuroprotective effects of neural stem cell exosomes after spinal cord injury *via* an miR-219a-2-3p/YY1 mechanism. Aging (Albany NY) 11, 12278–12294. 10.18632/aging.102568 31848325 PMC6949049

[B80] Mashouri L. Yousefi H. Aref A. R. Ahadi A. M. Molaei F. Alahari S. K. (2019). Exosomes: composition, biogenesis and mechanisms in cancer metastasis and drug resistance. Mol. Cancer 18:75. 10.1186/s12943-019-0991-5 30940145 PMC6444571

[B81] Mattick J. S. (2001). Non-coding RNAs: the architects of eukaryotic complexity. EMBO Rep. 2, 986–991. 10.1093/embo-reports/kve230 11713189 PMC1084129

[B82] McDonald J. W. Sadowsky C. (2002). Spinal-cord injury. Lancet 359, 417–425. 10.1016/S0140-6736(02)07603-1 11844532

[B83] Melo S. A. Luecke L. B. Kahlert C. Fernandez A. F. Gammon S. T. Kaye J.. (2015). Glypican-1 identifies cancer exosomes and detects early pancreatic cancer. Nature 523, 177–182. 10.1038/nature14581 26106858 PMC4825698

[B84] Mercer T. R. Dinger M. E. Mattick J. S. (2009). Long non-coding RNAs: insights into functions. Nat. Rev. Genet. 10, 155–159. 10.1038/nrg2521 19188922

[B85] Min Q. Parkinson D. B. Dun X. P. (2021). Migrating Schwann cells direct axon regeneration within the peripheral nerve bridge. Glia 69, 235–254. 10.1002/glia.23892 32697392

[B86] Nakazaki M. Morita T. Lankford K. L. Askenase P. W. Kocsis J. D. (2021). Small extracellular vesicles released by infused mesenchymal stromal cells target M2 macrophages and promote TGF-β upregulation, microvascular stabilization and functional recovery in a rodent model of severe spinal cord injury. J. Extracell. Vesicles 10:e12137. 10.1002/jev2.12137 34478241 PMC8408371

[B87] Nie H. Jiang Z. (2021). Bone mesenchymal stem cell-derived extracellular vesicles deliver microRNA-23b to alleviate spinal cord injury by targeting toll-like receptor TLR4 and inhibiting NF-κB pathway activation. Bioengineered 12, 8157–8172. 10.1080/21655979.2021.1977562 34663169 PMC8806461

[B88] Nikfarjam S. Rezaie J. Kashanchi F. Jafari R. (2020). Dexosomes as a cell-free vaccine for cancer immunotherapy. J. Exp. Clin. Cancer Res. 39:258. 10.1186/s13046-020-01781-x 33228747 PMC7686678

[B89] Noori L. Arabzadeh S. Mohamadi Y. Mojaverrostami S. Mokhtari T. Akbari M.. (2021). Intrathecal administration of the extracellular vesicles derived from human Wharton’s jelly stem cells inhibit inflammation and attenuate the activity of inflammasome complexes after spinal cord injury in rats. Neurosci. Res. 170, 87–98. 10.1016/j.neures.2020.07.011 32717259

[B90] Pan D. Li Y. Yang F. Lv Z. Zhu S. Shao Y.. (2021). Increasing toll-like receptor 2 on astrocytes induced by Schwann cell-derived exosomes promotes recovery by inhibiting CSPGs deposition after spinal cord injury. J. Neuroinflammation 18:172. 10.1186/s12974-021-02215-x 34372877 PMC8353762

[B91] Pant T. Juric M. Bosnjak Z. J. Dhanasekaran A. (2021). Recent insight on the non-coding RNAs in mesenchymal stem cell-derived exosomes: regulatory and therapeutic role in regenerative medicine and tissue engineering. Front. Cardiovasc. Med. 8:737512. 10.3389/fcvm.2021.737512 34660740 PMC8517144

[B92] Parolini I. Federici C. Raggi C. Lugini L. Palleschi S. De Milito A.. (2009). Microenvironmental pH is a key factor for exosome traffic in tumor cells. J. Biol. Chem. 284, 34211–34222. 10.1074/jbc.M109.041152 19801663 PMC2797191

[B94] Pomper N. Liu Y. Hoye M. L. Dougherty J. D. Miller T. M. (2020). CNS microRNA profiles: a database for cell type enriched microRNA expression across the mouse central nervous system. Sci. Rep. 10:4921. 10.1038/s41598-020-61307-5 32188880 PMC7080788

[B95] Quinzaños-Fresnedo J. Sahagún-Olmos R. C. (2015). [Micro RNA and its role in the pathophysiology of spinal cord injury - a further step towards neuroregenerative medicine]. Cir. Cir. 83, 442–447. 10.1016/j.circir.2015.05.045 26162489

[B96] Ramakonar H. Fehlings M. G. (2021). ’Time is Spine’: new evidence supports decompression within 24 h for acute spinal cord injury. Spinal Cord 59, 933–934. 10.1038/s41393-021-00654-0 34218264 PMC8338556

[B97] Ren Z. Qi Y. Sun S. Tao Y. Shi R. (2020). Mesenchymal stem cell-derived exosomes: hope for spinal cord injury repair. Stem Cells Dev. 29, 1467–1478. 10.1089/scd.2020.0133 33045910

[B98] Ren Z. W. Zhou J. G. Xiong Z. K. Zhu F. Z. Guo X. D. (2019). Effect of exosomes derived from MiR-133b-modified ADSCs on the recovery of neurological function after SCI. Eur. Rev. Med. Pharmacol. Sci. 23, 52–60. 10.26355/eurrev_201901_16747 30657546

[B99] Rong Y. Liu W. Wang J. Fan J. Luo Y. Li L.. (2019). Neural stem cell-derived small extracellular vesicles attenuate apoptosis and neuroinflammation after traumatic spinal cord injury by activating autophagy. Cell Death Dis. 10:340. 10.1038/s41419-019-1571-8 31000697 PMC6472377

[B100] Rowland J. W. Hawryluk G. W. Kwon B. Fehlings M. G. (2008). Current status of acute spinal cord injury pathophysiology and emerging therapies: promise on the horizon. Neurosurg. Focus 25:E2. 10.3171/FOC.2008.25.11.E2 18980476

[B101] Ryken T. C. Hurlbert R. J. Hadley M. N. Aarabi B. Dhall S. S. Gelb D. E.. (2013). The acute cardiopulmonary management of patients with cervical spinal cord injuries. Neurosurgery 72, 84–92. 10.1227/NEU.0b013e318276ee16 23417181

[B102] Sakamoto K. Ozaki T. Ko Y. C. Tsai C. F. Gong Y. Morozumi M.. (2019). Glycan sulfation patterns define autophagy flux at axon tip *via* PTPRσ-cortactin axis. Nat. Chem. Biol. 15, 699–709. 10.1038/s41589-019-0274-x 31061498

[B103] Sato-Kuwabara Y. Melo S. A. Soares F. A. Calin G. A. (2015). The fusion of two worlds: non-coding RNAs and extracellular vesicles–diagnostic and therapeutic implications (Review). Int. J. Oncol. 46, 17–27. 10.3892/ijo.2014.2712 25338714 PMC4238728

[B104] Segel M. Lash B. Song J. Ladha A. Liu C. C. Jin X.. (2021). Mammalian retrovirus-like protein PEG10 packages its own mRNA and can be pseudotyped for mRNA delivery. Science 373, 882–889. 10.1126/science.abg6155 34413232 PMC8431961

[B105] Shahzad U. Krumholtz S. Rutka J. T. Das S. (2021). Noncoding RNAs in glioblastoma: emerging biological concepts and potential therapeutic implications. Cancers (Basel) 13:1555. 10.3390/cancers13071555 33800703 PMC8037102

[B107] Shao M. Jin M. Xu S. Zheng C. Zhu W. Ma X.. (2020). Exosomes from long noncoding RNA-Gm37494-ADSCs repair spinal cord injury *via* shifting microglial M1/M2 polarization. Inflammation 43, 1536–1547. 10.1007/s10753-020-01230-z 32307615

[B106] Shao A. Tu S. Lu J. Zhang J. (2019). Crosstalk between stem cell and spinal cord injury: pathophysiology and treatment strategies. Stem Cell Res. Ther. 10:238. 10.1186/s13287-019-1357-z 31387621 PMC6683526

[B108] Sheng X. Zhao J. Li M. Xu Y. Zhou Y. Xu J.. (2021). Bone marrow mesenchymal stem cell-derived exosomes accelerate functional recovery after spinal cord injury by promoting the phagocytosis of macrophages to clean myelin debris. Front. Cell. Dev. Biol. 9:772205. 10.3389/fcell.2021.772205 34820385 PMC8606563

[B109] Shi Z. Pan B. Feng S. (2018). The emerging role of long non-coding RNA in spinal cord injury. J. Cell. Mol. Med. 22, 2055–2061. 10.1111/jcmm.13515 29392896 PMC5867120

[B110] Silver J. Miller J. H. (2004). Regeneration beyond the glial scar. Nat. Rev. Neurosci. 5, 146–156. 10.1038/nrn1326 14735117

[B111] Smyth T. Kullberg M. Malik N. Smith-Jones P. Graner M. W. Anchordoquy T. J. (2015). Biodistribution and delivery efficiency of unmodified tumor-derived exosomes. J. Control. Release 199, 145–155. 10.1016/j.jconrel.2014.12.013 25523519 PMC4441346

[B112] Sun G. Li G. Li D. Huang W. Zhang R. Zhang H.. (2018). hucMSC derived exosomes promote functional recovery in spinal cord injury mice *via* attenuating inflammation. Mater. Sci. Eng. C Mater. Biol. Appl. 89, 194–204. 10.1016/j.msec.2018.04.006 29752089

[B113] Tator C. H. (1995). Update on the pathophysiology and pathology of acute spinal cord injury. Brain Pathol. 5, 407–413. 10.1111/j.1750-3639.1995.tb00619.x 8974623

[B114] Thakur B. K. Zhang H. Becker A. Matei I. Huang Y. Costa-Silva B.. (2014). Double-stranded DNA in exosomes: a novel biomarker in cancer detection. Cell Res. 24, 766–769. 10.1038/cr.2014.44 24710597 PMC4042169

[B115] Thery C. Witwer K. W. Aikawa E. Alcaraz M. J. Anderson J. D. Andriantsitohaina R.. (2018). Minimal information for studies of extracellular vesicles 2018 (MISEV2018): a position statement of the International Society for Extracellular Vesicles and update of the MISEV2014 guidelines. J. Extracell. Vesicles 7:1535750. 10.1080/20013078.2018.1535750 30637094 PMC6322352

[B116] Thery C. Zitvogel L. Amigorena S. (2002). Exosomes: composition, biogenesis and function. Nat. Rev. Immunol. 2, 569–579. 10.1038/nri855 12154376

[B117] Todor D. R. Mu H. T. Milhorat T. H. (2000). Pain and syringomyelia: a review. Neurosurg. Focus 8:E11. 10.3171/foc.2000.8.3.11 16676923

[B118] Tran A. P. Warren P. M. Silver J. (2021). New insights into glial scar formation after spinal cord injury. Cell Tissue Res. 10.1007/s00441-021-03477-w. [Online ahead of print]. 34076775 PMC8975767

[B119] Treiber T. Treiber N. Meister G. (2019). Regulation of microRNA biogenesis and its crosstalk with other cellular pathways. Nat. Rev. Mol. Cell Biol. 20, 5–20. 10.1038/s41580-018-0059-1 30728477

[B120] Tsarouchas T. M. Wehner D. Cavone L. Munir T. Keatinge M. Lambertus M.. (2018). Dynamic control of proinflammatory cytokines Il-1beta and Tnf-alpha by macrophages in zebrafish spinal cord regeneration. Nat. Commun. 9:4670. 10.1038/s41467-018-07036-w 30405119 PMC6220182

[B121] Tu Y. K. Hsueh Y. H. (2020). Extracellular vesicles isolated from human olfactory ensheathing cells enhance the viability of neural progenitor cells. Neurol. Res. 42, 959–967. 10.1080/01616412.2020.1794371 32700620

[B122] van der Scheer J. W. Goosey-Tolfrey V. L. Valentino S. E. Davis G. M. Ho C. H. (2021). Functional electrical stimulation cycling exercise after spinal cord injury: a systematic review of health and fitness-related outcomes. J. Neuroeng. Rehabil. 18:99. 10.1186/s12984-021-00882-8 34118958 PMC8196442

[B123] van Niel G. D’Angelo G. Raposo G. (2018). Shedding light on the cell biology of extracellular vesicles. Nat. Rev. Mol. Cell Biol. 19, 213–228. 10.1038/nrm.2017.125 29339798

[B124] Vietri M. Radulovic M. Stenmark H. (2020). The many functions of ESCRTs. Nat. Rev. Mol. Cell Biol. 21, 25–42. 10.1038/s41580-019-0177-4 31705132

[B125] Vogel A. Upadhya R. Shetty A. K. (2018). Neural stem cell derived extracellular vesicles: attributes and prospects for treating neurodegenerative disorders. EBioMedicine 38, 273–282. 10.1016/j.ebiom.2018.11.026 30472088 PMC6306394

[B130] Wang Y. Lai X. Wu D. Liu B. Wang N. Rong L. (2021). Umbilical mesenchymal stem cell-derived exosomes facilitate spinal cord functional recovery through the miR-199a-3p/145-5p-mediated NGF/TrkA signaling pathway in rats. Stem Cell Res. Ther. 12:117. 10.1186/s13287-021-02148-5 33579361 PMC7879635

[B129] Wang W. Z. Li J. Liu L. Zhang Z. D. Li M. X. Li Q.. (2021). Role of circular RNA expression in the pathological progression after spinal cord injury. Neural Regen. Res. 16, 2048–2055. 10.4103/1673-5374.308100 33642393 PMC8343338

[B127] Wang L. Pei S. Han L. Guo B. Li Y. Duan R.. (2018). Mesenchymal stem cell-derived exosomes reduce A1 astrocytes *via* downregulation of phosphorylated NFκB P65 subunit in spinal cord injury. Cell. Physiol. Biochem. 50, 1535–1559. 10.1159/000494652 30376671

[B126] Wang J. Rong Y. Ji C. Lv C. Jiang D. Ge X.. (2020). MicroRNA-421-3p-abundant small extracellular vesicles derived from M2 bone marrow-derived macrophages attenuate apoptosis and promote motor function recovery *via* inhibition of mTOR in spinal cord injury. J. Nanobiotechnol. 18:72. 10.1186/s12951-020-00630-5 32404105 PMC7222346

[B132] Webb R. L. Kaiser E. E. Scoville S. L. Thompson T. A. Fatima S. Pandya C.. (2018). Human neural stem cell extracellular vesicles improve tissue and functional recovery in the murine thromboembolic stroke model. Transl. Stroke Res. 9, 530–539. 10.1007/s12975-017-0599-2 29285679 PMC6132936

[B133] Whetstone W. D. Hsu J. Y. Eisenberg M. Werb Z. Noble-Haeusslein L. J. (2003). Blood-spinal cord barrier after spinal cord injury: relation to revascularization and wound healing. J. Neurosci. Res. 74, 227–239. 10.1002/jnr.10759 14515352 PMC2837839

[B134] Wu X. Wang L. Cong M. Shen M. He Q. Ding F.. (2020). Extracellular vesicles from skin precursor-derived Schwann cells promote axonal outgrowth and regeneration of motoneurons *via* Akt/mTOR/p70S6K pathway. Ann. Transl. Med. 8:1640. 10.21037/atm-20-5965 33490152 PMC7812244

[B135] Xiao X. Li W. Rong D. Xu Z. Zhang Z. Ye H.. (2021). Human umbilical cord mesenchymal stem cells-derived extracellular vesicles facilitate the repair of spinal cord injury *via* the miR-29b-3p/PTEN/Akt/mTOR axis. Cell Death Discov. 7:212. 10.1038/s41420-021-00572-3 34381025 PMC8357833

[B136] Xin W. Qiang S. Jianing D. Jiaming L. Fangqi L. Bin C.. (2021). Human bone marrow mesenchymal stem cell-derived exosomes attenuate blood-spinal cord barrier disruption *via* the TIMP2/MMP pathway after acute spinal cord injury. Mol. Neurobiol. 58, 6490–6504. 10.1007/s12035-021-02565-w 34554399

[B137] Xu G. Ao R. Zhi Z. Jia J. Yu B. (2019). miR-21 and miR-19b delivered by hMSC-derived EVs regulate the apoptosis and differentiation of neurons in patients with spinal cord injury. J. Cell. Physiol. 234, 10205–10217. 10.1002/jcp.27690 30387159

[B138] Yi H. Wang Y. (2021). A meta-analysis of exosome in the treatment of spinal cord injury. Open Med. (Wars) 16, 1043–1060. 10.1515/med-2021-0304 34307887 PMC8284334

[B141] Yu T. Zhao C. Hou S. Zhou W. Wang B. Chen Y. (2019). Exosomes secreted from miRNA-29b-modified mesenchymal stem cells repaired spinal cord injury in rats. Braz. J. Med. Biol. Res. 52:e8735. 10.1590/1414-431X20198735 31826179 PMC6903804

[B139] Yu B. Zhou S. Yi S. Gu X. (2015). The regulatory roles of non-coding RNAs in nerve injury and regeneration. Prog. Neurobiol. 134, 122–139. 10.1016/j.pneurobio.2015.09.006 26432164

[B142] Yuan X. Wu Q. Wang P. Jing Y. Yao H. Tang Y.. (2019). Exosomes derived from pericytes improve microcirculation and protect blood-spinal cord barrier after spinal cord injury in mice. Front. Neurosci. 13:319. 10.3389/fnins.2019.00319 31040762 PMC6476953

[B143] Zhang A. Bai Z. Yi W. Hu Z. Hao J. (2021). Overexpression of miR-338-5p in exosomes derived from mesenchymal stromal cells provides neuroprotective effects by the Cnr1/Rap1/Akt pathway after spinal cord injury in rats. Neurosci. Lett. 761:136124. 10.1016/j.neulet.2021.136124 34302891

[B144] Zhang B. Lin F. Dong J. Liu J. Ding Z. Xu J. (2021). Peripheral macrophage-derived exosomes promote repair after spinal cord injury by inducing local anti-inflammatory type microglial polarization *via* increasing autophagy. Int. J. Biol. Sci. 17, 1339–1352. 10.7150/ijbs.54302 33867850 PMC8040463

[B146] Zhang M. Wang L. Huang S. He X. (2021). Exosomes with high level of miR-181c from bone marrow-derived mesenchymal stem cells inhibit inflammation and apoptosis to alleviate spinal cord injury. J. Mol. Histol. 52, 301–311. 10.1007/s10735-020-09950-0 33548000

[B145] Zhang C. Zhang C. Xu Y. Li C. Cao Y. Li P. (2020). Exosomes derived from human placenta-derived mesenchymal stem cells improve neurologic function by promoting angiogenesis after spinal cord injury. Neurosci. Lett. 739:135399. 10.1016/j.neulet.2020.135399 32979457

[B147] Zhao C. Zhou X. Qiu J. Xin D. Li T. Chu X.. (2019). Exosomes derived from bone marrow mesenchymal stem cells inhibit complement activation in rats with spinal cord injury. Drug Des. Devel. Ther. 13, 3693–3704. 10.2147/DDDT.S209636 31695336 PMC6817353

[B148] Zhong D. Cao Y. Li C. J. Li M. Rong Z. J. Jiang L.. (2020). Neural stem cell-derived exosomes facilitate spinal cord functional recovery after injury by promoting angiogenesis. Exp. Biol. Med. (Maywood) 245, 54–65. 10.1177/1535370219895491 31903774 PMC6987743

[B151] Zhou X. Chu X. Yuan H. Qiu J. Zhao C. Xin D.. (2019). Mesenchymal stem cell derived EVs mediate neuroprotection after spinal cord injury in rats *via* the microRNA-215p/FasL gene axis. Biomed. Pharmacother. 115:108818. 10.1016/j.biopha.2019.108818 31102912

[B149] Zhou S. Ding F. Gu X. (2016). Non-coding RNAs as emerging regulators of neural injury responses and regeneration. Neurosci. Bull. 32, 253–264. 10.1007/s12264-016-0028-7 27037691 PMC5563772

[B150] Zhou W. Silva M. Feng C. Zhao S. Liu L. Li S.. (2021). Exosomes derived from human placental mesenchymal stem cells enhanced the recovery of spinal cord injury by activating endogenous neurogenesis. Stem Cell Res. Ther. 12:174. 10.1186/s13287-021-02248-2 33712072 PMC7953814

[B152] Zhou Y. Wen L. L. Li Y. F. Wu K. M. Duan R. R. Yao Y. B.. (2022). Exosomes derived from bone marrow mesenchymal stem cells protect the injured spinal cord by inhibiting pericyte pyroptosis. Neural Regen. Res. 17, 194–202. 10.4103/1673-5374.314323 34100456 PMC8451579

